# Research on the Difference Between Recreational Walking and Transport Walking Among the Elderly in Mega Cities With Different Density Zones: The Case of Guangzhou City

**DOI:** 10.3389/fpubh.2021.775103

**Published:** 2021-11-15

**Authors:** Peng Zang, Hualong Qiu, Fei Xian, Xiang Zhou, Shifa Ma, Yabo Zhao

**Affiliations:** Department of Architecture, Guangdong University of Technology, Guangzhou, China

**Keywords:** recreational walking, transport walking, built environment, elderly, Guangzhou

## Abstract

Walking is the easiest method of physical activity for older people, and current research has demonstrated that the built environment is differently associated with recreational and transport walking. This study modelled the environmental characteristics of three different building density zones in Guangzhou city at low, medium, and high densities, and examined the differences in walking among older people in the three zones. The International Physical Activity Questionnaire (IPAQ) was used to investigate the recreational and transport walking time of older people aged 65 years and above for the past week, for a total of three density zones (*N* = 597) and was analysed as a dependent variable. Geographic Information Systems (GIS) was used to identify 300, 500, 800, and 1,000 m buffers and to assess differences between recreational and transport walking in terms of the built environment [e.g., land-use mix, street connectivity, Normalised Difference Vegetation Index (NDVI) data]. The data were processed and validated using the SPSS software to calculate Pearson's correlation models and stepwise regression models between recreation and transit walking and the built environment. The results found that land use mix and NDVI were positively correlated with transport walking in low-density areas and that transport walking was negatively correlated with roadway mediated centrality (BtE) and Point-of-Interest (PoI) density. Moreover, recreational walking in medium density areas was negatively correlated with self-rated health, road intersection density, and PoI density while positively correlated with educational attainment, population density, land use mix, street connectivity, PoIs density, and NDVI. Transport walking was negatively correlated with land-use mix, number of road crossings while positively correlated with commercial PoI density. Street connectivity, road intersection density, DNVI, and recreational walking in high-density areas showed negative correlations. Moreover, the built environment of older people in Guangzhou differed between recreational and transport walking at different densities. The richness of PoIs has different effects on different types of walking.

## Background

### Ageing Trends

The problem of population ageing is becoming more and more serious worldwide, and the World Population Prospects 2019 programme predicts that the elderly population in China will reach 300 million by 2025, with the ageing rate exceeding 20% and entering a deeply ageing society. In 2019, China issued the National Medium and Long-term Plan for Actively Coping with Population Ageing and proposed the construction of a “high-quality service and product supply service system for the elderly,” with the home as the basis and the community as the backbone. During 2021, they saw the authorisation of the Xinhua News Agency to release the 14th Five-Year Plan (1), which clearly states in Chapter 45, Section 3, “[i]mproving the elderly care service system,” that the system of elderly care at home should be improved, the urban environment should be adapted to ageing, and the community elderly care system should be improved. According to the statistics released by the Guangzhou Municipal Health Commission (GMHC), by the end of 2019, the population aged 60 and above in Guangzhou was 1,755,100, with the ageing population accounting for 18.40% of the total household population GMHC (2). Guangzhou has already entered an ageing society, and the health conflicts caused by ageing are increasing. Therefore, how to improve the participation and level of daily physical activity of the elderly to maintain their physical health has become an important topic of research and a hot topic in many fields at home and abroad.

### Benefits of Walking

Inactive behaviours such as prolonged sitting are the cause of a high incidence of chronic diseases that threaten the health of the people (3, 4). Studies have shown that as the body ages, physical performance decreases, with maximum oxygen uptake decreasing by 8–16% for every decade after the age of 30 (5). Muscle strength decreases by 10–15% every year. Moderate physical activity has a positive impact on the health of older people, and physical activity is beneficial in reducing the incidence of chronic diseases and some sudden illnesses (6). Based on the results of epidemiological studies, it has been demonstrated that walking has health benefits for older people, with overall results similar to those of physical exercise.

In addition, a study has shown that an increase in walking time increases the maximum oxygen uptake of older people (7). More walking promotes cardiovascular health and may prevent associated cardiovascular chronic diseases. Walking increases aerobic capacity, reduces Body Mass Index (BMI) and body fat, and gradually improves diastolic blood pressure caused by prolonged sedentary time (8).

Overall, research has shown that regular walking not only has a positive impact on body fat and cardio-respiratory fitness, but that walking could also improve physical strength and flexibility. Given that declines in strength and flexibility could affect the independence of older people, this finding suggested that reasonably regular walking can help older people maintain their independence in life.

### Built Environment and Walking

Research on walking environments increasingly shows that walking in residential settings is influenced not only by population density and the social characteristics of the population (such as age, gender, and income) but also by the built environment around an own residence (9–13). Previous research has demonstrated that good accessibility to usable green space is positively associated with walking behaviour and that better green space environments motivate older people to walk (12, 14, 15). Meanwhile, recent studies show that street greening has a positive effect on walking propensity and time (16). Factors of the built environment include land use diversity, street connectivity, building density, and accessibility (17).

However, there are some studies that show inconsistent results for the correlation between factors of the environment and walking. The study of Thornton et al. (18) found a positive correlation between land use mix and walking, while the work of Zhang et al. (19) found a negative correlation, with inconsistent results likely due to differences in objective perceptions of the study sample (10). Other later studies have used Geographic Information Systems (ArcGIS) to analyse the sample sites by delineating a specific radius of 200 to 2,000 m with residents as the central point for environmental analysis, which more objectively evaluated environmental factors, and re-did the study between the subdivided environment and walking, obtaining results that were more different than those from the previous non-subdivided environment (20–22). Some research has explored the application of environmental relevance (e.g., land use mix, street connectivity) of autonomous openly provided geographic information (e.g., open street maps), but the accuracy and reliability of their geographic information still need to be validated (23, 24).

### Transport Walking and Recreational Walking

The impact of the built environment on walking also varies depending on the purpose of walking and can be broadly divided into two types of walking, namely, transport walks motivated by walking to a destination (e.g., to work, to an event), and recreational walks specifically for exercise or to a recreational area such as a park., both of which are physical activities beneficial to the health of an individual (10, 11, 25). Although there are some inconsistencies in the theoretical results, research suggests that transport walking is primarily promoted through road density, land use, and accessibility to public transport, while recreational walking is related to community neighbourhoods, community pavements, and the ease of access and number of entrances to parks and activity venues (10, 26). The walking behaviour of older people in high-density urban settings also varies depending on the time of day. Fewer studies have examined differences in the relationship between environmental and recreational and transport walking among older people in Guangzhou, China.

To fill these research gaps, this study investigated how different densities of the built environment affected recreational walking and transport walking in Guangzhou. As an old first-tier city in China, Guangzhou has a complex and diverse urban fabric, with a high-density old city, a low-density new city, and other medium-density areas. Our research questions were as follows:

1. How does the built environment differ in association with different types of walking at different densities in the same city?
2. Are differences in the built environment and demographic characteristics associated with differences in transport walking times and recreational walking times?

## Methods

### Research Design and Study Population

**Figure 1:**
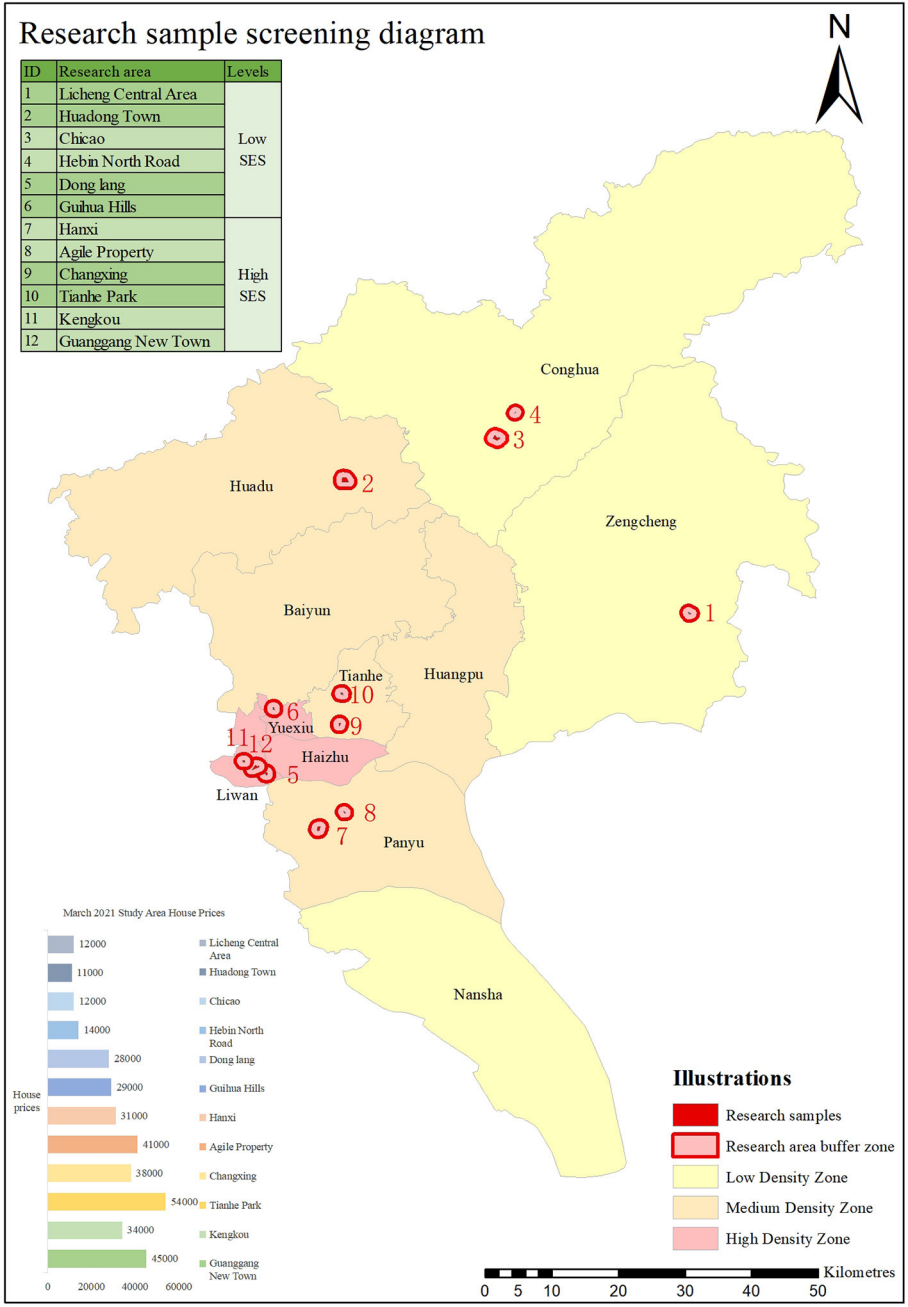
Research sample screening diagram.

This study was based on a survey of parks, selected to represent the surrounding community based on the different population density zoning of the Guangzhou city plan,^1^, where density zones 1, 2, and 3 corresponded to the low, medium, and high-density zones of this article. The areas were sampled according to the price of the area in March 2021 divided into low Socio-Economic Status (SES) and high SES, with areas below 30,000 RMB/m^2^ being low SES and areas >30,000 RMB/m^2^ being high SES, divided into six groups in total. We considered people aged 65–74, 74–84, 85, and over. Respondents were surveyed in the spring to avoid seasonal effects. The survey considered demographic characteristics (e.g., income, age, education) and travel information (e.g., broad questions related to the mode of transport, travel time). In order to maximise the sample size, 600 data were pooled and after excluding incomplete data records, our representative sample of older people in Guangzhou was 597 respondents in total (see Figure 1 below).

Our preliminary research of the 12 sample areas that we studied has revealed that the built environment varied significantly between the samples at different population densities in Guangzhou, so much so that during the initial modelling process it was found that the data for the built environment in the low-density areas significantly reduced the data for the more distinctive features of the built environment in the medium- and high-density areas. In order to prevent the model from overlooking some environmental features in its calculations, the low, medium, and high-density areas were modelled and calculated separately to ensure sufficient objectivity. A summary of the selected cases was shown in Table 1.

**Table 1:** Research regions.

**Low density**	**Medium density**	**High density**
Li Cheng Central/Huadong Town	Chicao/Hebin North Road	Donglang/Guihua Hills
Hanxi/Agile Property	Changxing/Tianhe Park	Kengkou/Guanggang New Town

### Walking Behaviour

The International Physical Activity Questionnaire-Long Version (IPAQ-LC) was used to group transport walking and recreational walking in the questionnaire based on the questionnaire and the purpose of travel from the interviews. The total daily duration of these two types of walking in minutes per person was used as the dependent variable. Additionally, areas outside the residential environment (>1,000 m) were excluded.

### Built Environment

In order to simulate the walking environment of older people, we applied buffers generated at the scale of the residential neighbourhood of the respondents. To incorporate the current walking environment, buffers of 300, 500, 800, and 1,000 m radius were used and compared (20, 27).

1. The normalized vegetation index (NDVI) (28) was used to visualize the amount of outdoor greenery. NDVI, which represented the chlorophyll content of the vegetation canopy (29), was obtained to extract the NDVI of Guangzhou City from remote sensing data with a resolution of 10 m accuracy in 2021. NDVI values ranged from 1 to −1, with higher NDVI values indicating more green and negative values corresponding to water bodies.
2. Land-use mixing was achieved through GIS calculations, which measured the type of use in qualitative residential settings where land was distributed (30). We obtained environmental data for the city of Guangzhou for the year 2021 from SkyMap and publicly available data from the Guangzhou Environmental Resources Bureau. Following previous studies by Bentley et al. (31) and Turrell et al. (32), we categorized the total land-use types in terms of the actual use by older people into seven categories, namely residential, recreational, commercial, educational, medical, and service. An index was calculated based on the proportion of area in each land use category. Its value ranges between 0 and 1, with higher values implying greater diversity.
3. Street connectivity was related to the design of the street layout (27). Higher street connectivity usually leads to more walking, while the opposite is true for more cul-de-sacs (10). Intersection data for this study were obtained from Sky Map data China (33) and calculated through GIS software.
4. BtE (mediated centrality) and MED (proximity centrality) were calculated in the software Spatial Design Network Analysis (sDNA^2^) for 300, 500, 800, and 1,000 m as control variables.

### Control Variables

We adjusted for several demographic and socio-economic characteristics of the respondents. Age was divided into three categories, which were 65–74, 75–84, and >85. Education level was divided into low (none), medium (primary education), and high (secondary and above). Self-rated health status was categorised as very good, fair, and bad.

### Statistical Analysis

Firstly, in order to ensure that the grouped studies were meaningful, a test of variance between groups was required, using SPSS to test for variance between the independent variables, as statistically significant between the independent variables under the three categories of the low, medium, and high (see Table 2 below).

**Table 2:** Tests for differences between groups in the low, medium, and high-density subgroups.

**Group**	**Age**	**Education level**	**Self-assessed health**	**Land use diversity**	**Street connectivity**	**Road intersection density**
Low density	–	1.97 ± 0.43^b^	1.93 ± 0.32^a^	0.39 ± 0.06^b^	1.88 ± 1.82a	63.11 ± 18.41^b^
Medium density	–	2.13 ± 0.46^a^	1.86 ± 0.47^a^	0.37 ± 0.15^b^	1.75 ± 0.07^b^	126.01 ± 55.01^a^
High density	–	2.00 ± 0.47^b^	1.72 ± 0.55^b^	0.60 ± 0.15^a^	1.67 ± 1.50^c^	129.25 ± 44.22^b^
	Betweeness centrality 300 n	Betweeness centrality 500 n	Betweeness centrality 800 n	Betweeness centrality 1,000 n	Inter mediation centrality 300 n	Inter mediation centrality 500 n
Low density	0.40 ± 0.12^a^	0.54 ± 0.05^b^	0.50 ± 0.03^b^	0.50 ± 0.02^b^	0.15 ± 0.04^a^	–
Medium density	0.44 ± 0.08^b^	0.51 ± 0.04^a^	0.47 ± 0.07^c^	0.50 ± 0.08^b^	0.12 ± 0.02^b^	–
High density	0.56 ± 0.09^c^	0.54 ± 0.04^b^	0.55 ± 0.06^a^	0.53 ± 0.06^a^	0.13 ± 0.04^b^	–
	Inter mediation centrality 800 n	Inter mediation centrality 1,000 n	Commercial PoI	Recreational PoI	Medical PoI	Education PoI
Low density	0.14 ± 0.05^a^	0.14 ± 0.04^a^	93.25 ± 61.23^b^	14.70 ± 8.53^b^	12.20 ± 8.43^c^	19.35 ± 11.70^b^
Medium density	0.12 ± 0.01^b^	0.12 ± 0.01^b^	201.50 ± 126.49^a^	25.30 ± 18.54^a^	27.25 ± 19.88^a^	63.69 ± 48.98^a^
High density	0.12 ± 0.05^b^	0.12 ± 0.05^b^	116.90 ± 86.78^b^	16.60 ± 8.70^b^	19.20 ± 10.01^b^	25.93 ± 17.55^b^
	Public Administration PoI	Number of Bus Stops	Distance to Bus Stops	Number of metro stations	Metro Station Distance	Number of overpasses
Low density	21.05 ± 17.22^c^	5.48 ± 3.38^b^	278.06 ± 214.12^a^	5.32 ± 4.43^b^	871.32 ± 549.95^a^	0 ± 0^c^
Medium density	56.30 ± 41.86^a^	25.97 ± 13.37^a^	236.36 ± 230.56^a^	2.25 ± 2.86^c^	372.49 ± 460.37^c^	1.75 ± 1.92^b^
High density	41.22 ± 27.77^b^	28.30 ± 7.50^a^	113.62 ± 53.65^b^	9.54 ± 7.91^a^	572.73 ± 274.41^b^	2.26 ± 1.65^a^
	No. of cut-off roads	NDVI				
Low density	72.25 ± 34.72^c^	0.45 ± 0.05^a^				
Medium density	106.90 ± 28.07^b^	0.43 ± 0.08^b^				
High density	131.09 ± 44.13^a^	0.34 ± 0.05^c^				

*Values with superscript letters a, b, and c are significantly different across columns (P < 0.05)*.

Descriptive statistics summary data. We checked for covariance between multiple different environmental variables using SPSS correlation tests and demonstrated no covariance in the data by VIF < 5. Walking time for recreational and transport was used as the dependent variable in our stepwise regression analysis (35, 36). Since some respondents reported only partial walking time, in correlation tests, we assessed the association between walking and environmental variables with buffer sizes of 300, 500, 800, and 1,000 m. The significance of each variable was assessed based on 95% CIs.

## Results

### Descriptive Statistics

Table 3 presents 597 valid questionnaires after removing invalid questionnaires, of which 86.6% were aged 65–74, 11.89% were aged 75–84 and 1.51% were aged 85 or above. The educational level of those with higher education was 8.71%, those with secondary education was 79.06%, and those with no education were 12.23%. Self-rated health was excellent at 20.44%, good at 75.54%, and bad at 4.02%. Respondents spent more total time walking for recreation than for transport, street connectivity gradually decreased with increasing density of the sample cities, road intersection density gradually increased with increasing urban density, the number of bus and metro stations increased step by step with increasing density of the sample cities, and NDVI decreased step by step with increasing density of the sample cities.

**Table 3:** Descriptive statistics to study the characteristics of the population and the natural and built environment of the living environment (*n* = 597).

	**Total**		**Low density**		**Medium density**		**High density**	
	**% per** **category**	**Mean** **(std. dev.)**	**% per category**	**Mean** **(std. dev.)**	**% per** **category**	**Mean** **(std. dev.)**	**% per** **category**	**Mean** **(std. dev.)**
Indicator	100		33.00		33.67		33.33	
**Age**								
65–74	86.60		87.31		83.08		89.45	
75–84	11.89		11.68		15.42		8.54	
85+	1.51		1.02		1.49		2.01	
**Education level**								
Medium and above	8.71		10.66		4.48		11.06	
Elementary education	79.06		81.73		77.61		77.89	
None	12.23		7.8		19.91		11.06	
**Self-assessed health**								
Very good/good	20.44		9.14		18.91		33.17	
General	75.54		88.83		76.12		61.81	
Not good/very bad	4.02		2.03		4.98		5.03	
Population density		1.24 (1.04)		0.62 (0.61)		0.95 (0.92)		2.13 (0.88)
Transport walking		413.27 (477.52)		413.89 (523.52)		394.22 (419.12)		443.21 (493.32)
Recreational walking		440.03 (439.58)		388.85 (409.64)		486.63 (439.81)		355.75 (332.59)
Land use diversity		0.46 (0.16)		0.39 (0.06)		0.37 (0.15)		0.60 (0.15)
Street connectivity		1.74 (0.18)		1.77 (0.18)		1.75 (0.07)		1.59 (0.15)
Road intersection density		106.33 (51.96)		63.11 (18.42)		126.01 (55.01)		129.25 (44.22)
Betweeness centrality 300 n		0.47 (0.12)		0.40 (0.12)		0.44 (0.08)		0.56 (0.09)
Betweeness centrality 500 n		0.53 (0.43)		0.54 (0.05)		0.51 (0.04)		0.54 (0.03)
Betweeness centrality 800 n		0.51 (0.06)		0.50 (0.03)		0.47 (0.07)		0.55 (0.06)
Betweeness centrality 1,000 n		0.51 (0.06)		0.50 (0.02)		0.50 (0.08)		0.53 (0.06)
Inter mediation centrality 300 n		0.13 (0.04)		0.15 (0.04)		0.12 (0.02)		0.13 (0.04)
Inter mediation centrality 500 n		0.13 (0.03)		0.13 (0.04)		0.13 (0.01)		0.13 (0.03)
Inter mediation centrality 800 n		0.12 (0.04)		0.14 (0.05)		0.12 (0.01)		0.12 (0.05)
Inter mediation centrality 1,000 n		0.13 (0.04)		0.14 (0.04)		0.12 (0.01)		0.12 (0.05)
Commercial PoI		137.58 (106.18)		93.25 (61.23)		201.50 (126.49)		116.90 (86.78)
Recreational PoI		18.90 (13.77)		14.70 (8.53)		25.29 (18.85)		16.60 (8.70)
Medical PoI		19.60 (15.07)		12.20 (8.43)		27.25 (19.88)		19.20 (10.01)
Education PoI		36.47 (36.55)		19.35 (11.70)		63.69 (48.95)		25.93 (17.55)
Public Administration PoI		39.65 (33.92)		21.05 (17.22)		56.30 (41.86)		41.33 (27.77)
Number of bus stops		19.98 (13.67)		5.48 (3.38)		25.97 (13.37)		28.30 (7.46)
Distance to bus stops		209.21 (196.82)		278.06 (214.12)		236.36 (230.56)		113.62 (53.65)
Number of metro stations		5.70 (6.24)		5.33 (4.43)		2.25 (2.86)		9.54 (7.91)
Metro station distance		603.84 (487.46)		871.32 (549.95)		372.49 (460.37)		572.73 (274.41)
Number of overpasses		1.34 (1.75)		_		1.75 (1.92)		2.2 6(1.65)
No. of cut-off roads		103.53 (43.46)		72.25 (34.72)		106.90 (28.07)		131.09 (44.13)
NDVI		0.41 (0.08)		0.44 (0.05)		0.43 (0.08)		0.34 (0.05)

### Regression Analysis

Table 4 shows the analysis between the independent variables in the low-density areas, wherein there was a correlation between the built environment characteristics and transport walking in the low-density areas, while land-use mix, number of bus stops, distance to the nearest bus stop, and NDVI were positively correlated with transport walking. Finally, transport walking was positively correlated with MED 300 m, BtE 300 m, BtE 1,000 m, while Commercial PoIs, Recreation PoIs Medical PoIs, Education PoIs, and Public Administration PoIs were negatively correlated. There was no significant correlation between recreational walking and built environment characteristics in any of the low-density areas.

**Table 4:** Pearson correlation analysis for low, medium, and high-density areas (*n* = 597).

**Pearson correlation analysis of recreational walking (RW) and transit walking (TW) in low density areas**															
	**300 m buffer**				**500 m buffer**				**800 m buffer**				**1,000 m buffer**			
	**RW**		**TW**		**RW**		**TW**		**RW**		**TW**		**RW**		**TW**	
	**R**	***p*-value**	**R**	***p*-value**	**R**	***p*-value**	**R**	***p*-value**	**R**	***p*-value**	**R**	***p*-value**	**R**	***p*-value**	**R**	***p*-value**
Land use diversity	−0.042	0.555	0.177^*^	**0.031**	−0.019	0.823	0.114	0.166	−0.094	0.262	0.062	0.448	−0.064	0.445	0.094	0.252
Betweeness centrality 300 n	−0.001	0.993	−0.188^*^	**0.021**	−0.076	0.366	−0.188^*^	**0.021**	−0.076	0.366	−0.188^*^	**0.021**	−0.076	0.366	−0.188^*^	**0.021**
Intermediary centrality 300 n	0.033	0.646	−0.180^*^	**0.028**	−0.036	0.667	−0.180^*^	**0.028**	−0.036	0.667	−0.180^*^	**0.028**	−0.037	0.665	−0.180^*^	**0.028**
Intermediary centrality 1,000 n	–	–	–	–	–	–	–	–	–	–	–	–	−0.042	0.617	−0.166^*^	**0.042**
Commercial PoI	0.053	0.456	−0.179^*^	**0.029**	−0.014	0.867	−0.169^*^	**0.038**	0.001	0.99	−0.169^*^	**0.039**	0.020	0.815	−0.156	0.056
Recreational PoI	0.033	0.647	−0.161^*^	**0.049**	−0.004	0.965	−0.165^*^	**0.044**	0.016	0.845	−0.159	0.053	0.057	0.496	−0.116	0.157
Medical PoI	0.008	0.907	−0.183^*^	**0.025**	−0.015	0.857	−0.165^*^	**0.044**	0.000	0.996	−0.162^*^	0.047	0.02	0.812	−0.145	0.077
Education PoI	0.026	0.976	−0.183^*^	**0.025**	0.018	0.833	−0.141	0.086	0.017	0.836	−0.152	0.063	0.032	0.705	−0.145	0.076
Public Administration PoI	0.028	0.691	−0.160	0.051	−0.024	0.774	−0.171^*^	**0.036**	−0.024	0.778	−0.178^*^	**0.030**	0.010	0.904	−0.134	0.102
Number of Bus Stops	−0.064	0.373	0.147	0.074	−0.019	0.822	0.153	0.062	0.037	0.661	0.188^*^	**0.021**	0.057	0.5	0.183^*^	**0.025**
Distance to Bus Stops	−0.046	0.517	0.170^*^	**0.038**	0.014	0.865	0.170^*^	**0.038**	0.050	0.556	0.067	0.415	0.05	0.556	0.067	0.415
NDVI	0.000	0.996	0.073	0.372	0.002	0.981	0.083	0.331	0.004	0.996	0.127	0.122	−0.004	0.962	0.127^*^	**0.035**
**Pearson correlation analysis of recreational walking (RW) and transit walking (TW) in medium density areas**																
Education level	0.169^*^	**0.037**	0.214	0.214	0.169^*^	**0.037**	−0.095	0.214	0.169^*^	**0.037**	−0.095	0.214	0.169^*^	**0.037**	−0.095	0.214
Self-report health	−0.211^**^	**0.009**	−0.112	0.145	−0.211^**^	**0.009**	−0.112	0.145	−0.211^**^	**0.009**	−0.112	0.145	−0.211^**^	**0.009**	−0.112	0.145
Population density	0.291^**^	**0.000**	−0.067	0.381	0.291^**^	**0**	−0.067	0.381	0.291^**^	**0.000**	−0.067	0.381	0.291^**^	**0.000**	−0.067	0.381
Land use diversity	0.004	0.959	−0.166^*^	**0.030**	0.166^*^	**0.04**	−0.094	0.220	0.222^**^	**0.006**	−0.114	0.138	0.203^*^	**0.012**	−0.126	0.101
Street connectivity	0.227^**^	**0.005**	0.07	0.363	0.052	0.523	0.157^*^	0.04	0.053	0.51	−0.119	0.119	0.320^**^	**0.000**	−0.028	0.715
Road intersection density	−0.320^**^	**0.000**	−0.032	0.681	−0.258^**^	**0.001**	−0.04	0.605	−0.196^*^	**0.015**	−0.009	0.902	−0.108	0.181	−0.042	0.588
Betweeness centrality 300 n	0.04	0.621	0.162^*^	**0.034**	0.04	0.621	0.162^*^	0.034	0.04	0.621	0.162^*^	**0.034**	0.040	0.620	0.162^*^	**0.034**
Betweeness centrality 500 n	–	–	–	–	−0.208^**^	**0.01**	−0.128	0.093	−0.208^**^	**0.010**	−0.128	0.093	−0.207^*^	**0.010**	−0.13	0.089
Intermediary centrality 300 n	−0.036	0.659	0.102	0.182	−0.036	−0.068	0.105	0.169	−0.036	0.659	0.105	0.169	−0.034	0.677	0.104	0.175
Intermediation centrality 500 n	–	–	–	–	−0.324^**^	**0.000**	−0.009	0.903	−0.324^**^	**0.000**	−0.009	0.903	−0.323^**^	**0.000**	−0.009	0.909
Intermediary centrality 1000 n	–	–	–	–	–	–	–	–	–	–	–	–	−0.187^*^	**0.020**	0.083	0.279
Commercial PoI	−0.035	0.699	−0.178^*^	0.039	0.053	0.512	−0.128	0.094	0.092	0.255	−0.071	0.358	0.140	0.083	−0.031	0.685
Recreational PoI	0.146	0.106	−0.16	0.065	0.315^**^	**0.000**	0.112	0.199	0.260^**^	**0.004**	0.139	0.11	0.203^*^	**0.012**	0.004	0.959
Medical PoI	−0.311^**^	**0.000**	−0.114	0.189	−0.358^**^	**0.000**	0.048	0.585	−0.248^**^	**0.006**	0.126	0.147	−0.057	0.485	0.007	0.932
Education PoI	0.141	0.082	0.001	0.988	0.125	0.122	−0.009	0.907	0.177^*^	**0.028**	0.008	0.922	0.183^*^	**0.023**	0.014	0.856
Public Administration PoI	−0.381^**^	**0.000**	0.002	0.98	−0.173^*^	**0.032**	−0.007	0.928	−0.137	0.090	−0.011	0.881	−0.096	0.237	0	0.995
Number of Bus Stops	−0.148	0.066	−0.049	0.526	−0.229^**^	**0.004**	−0.040	0.600	−0.095	0.244	0.009	0.904	−0.044	0.586	−0.001	0.993
Number of metro stations	0.221^**^	**0.006**	0.077	0.313	0.221^**^	**0.006**	0.077	0.313	0.221^**^	0.006	0.077	0.313	0.269^**^	**0.001**	0.036	0.635
Metro Station Distance	0.221^**^	**0.006**	0.077	0.313	0.221^**^	**0.006**	0.077	0.313	0.221^**^	0.006	0.077	0.313	0.187^*^	**0.02**	–−0.143	0.062
Number of overpasses	0.086	0.291	0.082	0.285	0.200^*^	**0.013**	0.029	0.710	0.209^**^	**0.009**	0.043	0.574	0.213^**^	**0.008**	0.051	0.507
No. of cut-off roads	0.083	0.305	0.164^*^	**0.031**	−0.105	0.194	0.117	0.127	0.154	0.057	0.112	0.144	−0.127	0.116	−0.151^*^	**0.048**
NDVI	0.215^**^	**0.007**	0.067	0.384	0.174^*^	**0.031**	0.025	0.745	0.174^*^	**0.031**	0.025	0.745	0.115	0.157	0.122	0.112
**Pearson correlation analysis of recreational walking (RW) and transit walking (TW) in high density areas**																
Street connectivity	−0.131	0.126	−0.076	0.343	−0.219^**^	**0.009**	0.056	0.485	−0.251^**^	**0.003**	0.045	0.575	0.187^*^	**0.028**	0.09	0.261
Road intersection density	−0.146	0.087	0.058	0.469	−0.271^**^	**0.001**	0.006	0.935	−0.106	0.214	0.094	0.239	−0.266^**^	**0.0.006**	0.046	0.561
Betweeness centrality 300 n	−0.197^*^	**0.020**	−0.018	0.827	−0.197^*^	**−0.083**	−0.018	0.827	−0.197^*^	**0.020**	−0.018	0.827	−0.198^*^	**0.019**	−0.017	0.828
Number of Bus Stops	−0.201^*^	**0.018**	0.084	0.294	0.955	0.929	−0.015	0.849	−0.031	0.721	0.114	0.153	−0.128	0.132	0.081	0.307
Distance to Bus Stops	0.227^**^	**0.007**	−0.054	0.496	0.227^**^	**0.007**	−0.054	0.496	0.227^**^	**0.007**	−0.054	0.496	0.227^**^	0.007	−0.054	0.496
NDVI	0.115	0.157	0.122	0.112	−0171^*^	**0.044**	0.007	0.935	−0.147	0.084	0.038	0.638	0.060	0.482	0.105	0.187

* *p < 0.05*;

** *p < 0.01. Bold values represent having relevance*.

Changes in the low-density buffer also influenced the relationship between the built environment and transport walking, with land-use diversity (*R* = 0.117^*^; *P* = 0.031), showing a positive correlation only in the 300 m buffer while no positive correlation found in the 500, 800, and 1,000 m buffers. Education PoIs (*R* = −0.183^*^; *P* = 0.025) showed a negative correlation only in the 300 m buffer while no negative correlation was found in the 500, 800, and 1,000 m buffers. Recreation PoI (*R* = −0.161^*^; *P* = 0.049, *R* = −0.165^*^; *P* = 0.044) and Recreational PoIs (*R* = −0.183^*^; *P* = 0.025, *R* = −0.165^*^; *P* = 0.044) were negatively correlated in the 300 and 500 m buffers, while no negative correlation was found in the 800 and 1,000 m buffer. Public Administration PoIs (*R* = −0.171^*^; *P* = 0.036, *R* = −0.178^*^; *P* = 0.03) were negatively correlated between the 500 and 800 m buffers, while no negative correlation was found between the 300 and 1,000 m buffers. Commercial PoIs (*R* = −0.179^*^; *P* = 0.029, *R* = −0.169^*^; *P* = 0.038, *R* = −0.169^*^; *P* = 0.039) did not show a negative correlation only in the 1,000 m buffer. The number of bus stops (*R* = 0.188^*^; *P* = 0.021, *R* = 0.183^*^; *P* = 0.025) was positively correlated between the 800 and 1,000 m buffers, while not between the 300 and 500 m buffers. The nearest bus stop distance (*R* = 0.17^*^; *P* = 0.038, *R* = 0.17^*^; *P* = 0.038) showed a positive correlation with the 300 and 500 m buffers, while no positive correlation with the 800 and 1,000 m buffers. NDVI (*R* = 0.127^*^; *P* = 0.035) showed a positive correlation with the 1,000 m buffer only, while no positive correlation with the 300, 500, and 800 m buffers. Finally, 500 and 800 m buffers did not show a positive correlation.

Transport walking was negatively correlated with land-use diversity with number of 1,000 m buffer cut-off roads, while positively correlated with MED 300 m, Commercial PoI, with number of 300 m buffer cut-off roads. The correlations between buffers at medium densities also varied significantly, with transport walking being negatively correlated with land-use diversity (*R* = −0.166^*^; *P* = 0.03) and Commercial PoIs (*R* = −0.178^*^; *P* = 0.039) at the 300 m buffer, while no negative correlation at the 500, 800, and 1,000 m buffers, with number of cut-off roads (*R* = 0.164^*^; *P* = 0.031) showed a positive correlation, but the number of cut-off roads under the 1,000 m buffer (*R* = −0.151^*^; *P* = 0.048) showed a negative correlation while the 500 and 1,000 m buffers did not show a correlation.

Recreational walking was positively correlated with street connectivity under the 300 m buffer vs. the 1,000 m buffer (*R* = 0.227^**^; *P* = 0.005, *R* = 0.32^*^; *P* = 0.000), while the 500 and 800 m buffers did not show a positive correlation. There was no positive correlation with road intersection density under the 300, 500, and 800 m buffers (*R* = −0.32^**^; *P* = 0.000, *R* = −0.258^**^; *P* = 0.001, *R* = −0.196^*^; *P* = 0.015), while Medical PoIs (*R* = −0.311^**^; *P* = 0.000, *R* = −0.358^**^; *P* = 0.000, *R* = −0.248^**^; *P* = 0.006) were negatively correlated and the 1,000 m buffer showed no negative correlation. Positive correlations were found with Recreational PoIs for the 500, 800, and 1,000 m buffers (*R* = 0.315^**^; *P* = 0.000, *R* = 0.26^**^; *P* = 0.004, *R* = 0.203^*^; *P* = 0.012) while no positive correlations were found for the 300 m buffer. Positive correlation was found with Education PoIs under 800 and 1,000 m buffers (*R* = 0.177^*^; *P* = 0.028, *R* = 0.183^*^; *P* = 0.023) while there was no positive correlation with 300 and 500 m buffers. There was a negative correlation with Public Administration PoIs under the 300 and 500 m buffers (*R* = −0.381^**^; *P* = 0.000, *R* = −0.173^**^; *P* = 0.032) while there was no negative correlation with 800 and 1,000 m. There was a negative correlation with the number of buses stops under the 500 m buffer (*R* = −0.229^**^; *P* = 0.004) and no negative correlation with the other range buffers. Positive correlation with NDVI under 300, 500, and 800 m buffers (*R* = 0.215^**^; *P* = 0.007, *R* = 0.174^*^; *P* = 0.031, *R* = 0.174^*^; *P* = 0.031) while no positive correlation was presented for 1,000 m buffers.

Table 4 also shows the analysis between independent variables in high-density areas, wherein there was a correlation between environmental characteristics recreational walking in high-density areas, with street connectivity, road intersection density, MED 300 m, number of bus stops, and DNVI showing a negative correlation with recreational walking. There was a positive correlation with the distance to the nearest bus stop. Finally, no significant correlations were found between transport walking and built environment features in high-density areas.

There were also significant differences in the correlation between environmental architectural features and recreational walking in high-density buffer zones of different sizes, with street connectivity (*R* = −0.219^**^; *P* = 0.009, *R* = −0.251^**^; *P* = 0.003) being negatively correlated with recreational walking in the 300 and 500 m buffers, while street connectivity (*R* = 0.187^*^; *P* = 0.028) was positively correlated in 1,000 m. The correlation was positive in the 1,000 m buffer and did not show a correlation in the 300 m buffer. Road intersection density (*R* = −0.271^**^; *P* = 0.001, *R* = −0.266^**^; *P* = 0.006) was negatively correlated with recreational walking in the 500 and 1,000 m buffers, but not in the 300 and 800 m buffers. The number of bus stops (*R* = −0.201^*^; *P* = 0.018) was negatively correlated with recreational walking within the 300 m buffer while no negative correlation was observed within the 500, 800, and 1,000 m buffers.

### Regression Analysis

Table 5 summarises the stepwise regression results for recreational walking and transport walking at low, medium, and high densities. In particular, at low densities, there was no correlation between recreational walking and built and natural environment features, and within the 300, 500, and 1,000 m buffers, transport walking was negatively influenced by a regression value of −0.197^*^ (*t* = −2.450; *p* = 0.015 < 0.05) with MED 300 m, and the number of buses stops regression value of.195^*^ (*t* = 2.429; *p* = 0.016 < 0.05) had a positive influence relationship with transport walking.

**Table 5:** Statistical results of stepwise regression models for recreational walking (RW) and transport walking (TW) at low densities (*n* = 197).

	**300 m buffer**				**500 m buffer**				**800 m buffer**				**1,000 m buffer**			
	**RW**		**TW**		**RW**		**TW**		**RW**		**TW**		**RW**		**TW**	
	**β(95% CI)**	***p*-value**	**β(95% CI)**	***p*-value**	**β(95% CI)**	***p*-value**	**β(95% CI)**	***p*-value**	**β(95% CI)**	***p*-value**	**β(95% CI)**	***p*-value**	**β(95% CI)**	***p*-value**	**β(95% CI)**	***p*-value**
Betweeness centrality 300 n	–	–	–**0.197**	0.015^*^	–	–	–**0.197**	0.015^*^	–	–	−0.114	0.622	–	–	–**0.197**	0.015^*^
Number of Bus Stops	–	–	0.053	0.619	–	–	0.057	0.616	–	–	**0.195**	0.016^*^	–	–	−0.007	0.976
Statistical results of stepwise regression models for recreational walking (RW) and transport walking (TW) at medium density (n = 201).																
Self–report health	–**0.191**	0.000^**^	−0.125	0.127	–**0191**	0.013^*^	−0.118	0.118	–**0191**	0.013^*^	−0.118	0.118	–**0191**	0.013^*^	−0.119	0.117
Betweeness centrality 300 n	0.012	0.871	−0.109	0.699	0.012	0.871	**0.167**	0.028^*^	0.012	0.871	**0.167**	0.028^*^	0.015	0.841	**0.167**	0.028^*^
Intermediation centrality 500 n	–	–	–	–	–**0.368**	0.000^**^	0.029	0.708	–**0.312**	0.000^**^	0.029	0.708	–**0.311**	0.000^**^	0.026	0.733
Commercial PoI	−0.008	0.917	–**0.182**	0.017^*^	−0.017	0.821	0.065	0.685	−0.022	0.790	0.052	0.591	−0.029	0.743	0.041	0.624
Statistical results of stepwise regression models for recreational walking (RW) and transport walking (TW) at high densities (n = 200).																
Street connectivity	−0.103	0.220	–	–	−0.083	0.43	–	–	–**0.251**	0.003^**^	–	–	0.086	0.349	–	–
Road intersection density	0.104	0.440	–	–	–**0.271**	0.001^**^	–	–	0.178	0.150	–	–	–**0.266**	0.002^**^	–	–
NDVI	–**0.331**	0.001^**^	–	–	0.116	0.390	–	–	−0.087	0.310	–	–	0.079	0.342	–	–

* *p < 0.05*;

** *p < 0.01. Bold values represent having relevance*.

For the medium density areas, the BtE 500 m regression coefficient of −0.312^**^ (*t* = −4.122; *p* = 0.000 < 0.01) for the 500, 800, and 1,000 m buffers and the regression coefficient of −0.191^*^ (*t* = −2.522; *p* = 0.013 < 0.05) for the health self-report within all buffers were negatively associated with recreational walking. The MED 300 m regression coefficient of.167^*^ (*t* = 2.209; *p* = 0.028 < 0.05) within the 500, 800, and 1,000 m buffers had a positive impact relationship with transport walking. The Commercial PoIs regression coefficient of −0.182^*^ (*t* = −2.410; *p* = 0.017 < 0.05) within the 300 m buffer had a negative relationship with transport walking.

Transport walking at high densities was not correlated with built and natural environment features, and recreational walking was negatively associated with NDVI regression values of −0.237^**^ (*t* = −2.851; *p* = 0.005 < 0.01) within the 300 m buffer, intersection density regression values of −0.271^**^ (*t* = −3.299; *p* = 0.001 < 0.01) and −0.251^**^ (*t* = −3.036; *p* = 0.003 < 0.01) for the regression value of street connectivity within the 800 m buffer had a negative effect.

## Discussion

### Key Findings

This study examined the correlation and consistency between built and natural environment features and walking in megacities at different density buffers. Previous studies have demonstrated a positive correlation between land-use diversity and transport walking (18, 31), however in this study the situation was different at different densities, with land-use diversity only positively correlated with transport walking at low densities, land use diversity not encouraging more transport walking at medium densities but weakening, and no correlation at higher densities. At medium densities, land-use mix encouraged more recreational walking, but at low and high densities there was no such relationship. This phenomenon might be due to the fact that low-density areas are sparsely populated with a land-use mix that is most attractive for transit walking, while medium-density areas have a higher distribution of all types of PoIs compared with low-density areas resulting in a land-use diversity that is not more attractive to older people for transit walking and less demand for transit trips. However, the abundance of PoIs in medium-density results in a land-use diversity that is more attractive to seniors for recreational walking. The reason why high-density land-use diversity does not correlate with walking may be that high-density PoIs are dense enough that seniors tend to be able to reach the functional areas they need to meet their daily needs on the ground floor of their own residence. Therefore, there was no correlation with walking. The finding that NDVI was positively associated with transport walking at low densities and with recreational walking at medium densities was consistent with previous studies, but the finding that NDVI was negatively associated with recreational walking at high densities differed from previous studies (37–39). This phenomenon could be explained by the fact that recreation sites (e.g., pocket parks) with poorer environments lacking shelter space in high-density areas are located in areas with higher levels of vegetation, resulting in less recreational walking (40). We found that street connectivity and road crossing density were negatively correlated with recreational walking in small buffer sizes at high densities but positively correlated with medium density areas at high densities with buffers of 1,000 m, which is not consistent with previous studies (18). This could be explained by the fact that higher street connectivity in small areas at high densities may reduce the perceptions of safety of people or due to individual differences in perceptions of the neighbourhood, and secondly due to the fact that people are reluctant to walk in unsafe and uncomfortable areas due to the density of motorways (21, 41, 42).

We added MED and BtE to the independent variables of the built environment, where MED tended to respond to the importance of the global location and BtE to the centre of gravity of a small area, i.e., the density of the network. The results showed that at low densities MED 300, BtE 300, and BtE 1,000 m were all negatively correlated with transport walking. This phenomenon could be explained by the fact that at low densities the road network was relatively sparse, and the most important global zones were in residential areas, the areas with the highest network densities were adjacent to roads where the elderly lack a sense of security and therefore could lead to less transport walking. However, at medium densities, the MED 300 m was positively correlated with transport, which could be explained by the fact that the most important sites in the global location overlap with commercial sites, thus promoting transport walking for older people. Furthermore. 300 m BtE and 1,000 m BtE were negatively correlated with transport walking, as is the case at low densities where the network density was also adjacent to a motorway, resulting in a negative impact on transport walking. At higher densities, MED 300 m was negatively correlated with recreational walking. The high-density core areas were not the type of parks that seniors would choose for recreation, resulting in the MED having a negative effect on recreational walking for seniors.

Although the indices of the built environment at different densities were relatively consistent across buffer sizes, we found that the correlation for some environmental factors varied by geographic scale, which was consistent with the findings of Etman et al. (43) and Zang et al. (15). For example, land use diversity was positively correlated with transport walking in the low-density 300 m buffer zone, while no correlation was found at 500, 800, and 1,000 m. In contrast, in the medium-density zone at 500, 800, and 1,000 m, there was no correlation with transport walking. In contrast, transport walking was negatively correlated with land-use diversity in the medium density areas at 500, 800, and 1,000 m buffers, with no correlation found in the smaller 300 m buffers. No correlation between transport walking and land use diversity was found in any of the buffers in the high-density areas. This suggested that older people preferred to transport walking in areas of moderate density and that there was little need to walk for transport when densities were high enough. Furthermore, for other reasons such as time constraints and physical limitations, seniors chose not to walk long distances for transportation. In low-density areas, 500 and 1,000 m buffer zones transit walking was positively correlated with the number of bus stops, and 300 and 500 m buffer zones transit walking was positively correlated with the closest distance to a bus stop. Recreational walking in the medium density 500 m buffer was negatively correlated with the number of bus stops. High density 300 m buffers had a negative correlation between recreational walking and the number of bus stops, but all buffers of the same size have a positive correlation with the closest distance to a bus stop. The number of metro stops and the distance to the nearest metro stop were not correlated with walking in the low- and high-density areas, while the number of metro stops and the distance to the nearest metro stop were positively correlated with recreational walking in the medium-density areas for all buffer sizes. The remaining density areas transit stations and walking were not correlated across buffer sizes. In the lower density areas, the number of metro stations was low and the demand for transport was mainly by bus, which was consistent with the current study. At medium densities, the metro coverage was already more comprehensive and due to time constraints, the elderly rarely chose public transport as a transport option so no correlation was presented. At high densities, due to the fact that the density was sufficiently high, even though the metro network coverage was dense enough, it did not affect the mode of travel of the elderly, and the bus was more convenient than the metro, and it was only a short walk to the entertainment venues. The results of this study were currently different (44).

### Advantages and Limitations

This study has several strengths. Firstly, we used the most recent geographical sample of Guangzhou as a reference, the questionnaire and geographical data were collected more recently and are more up to date. Secondly, unlike previous studies on environmental perception (41, 45), we used GIS for more objective measurements and calculations of the built environment. Thirdly we distinguish between multiple buffer-sized environmental scales at different densities to examine the impact of multiple geographical scales on recreational walking and transport walking, a study that has rarely been used before.

There were also some shortcomings in that the volume of our data could have been further improved, and there was a lack of additional data on human subjects to minimise the objective effects of respondents. Secondly, our current study was limited to a walking study of older people in Guangzhou, Guangdong Province, China (a mega-city with extremely high building and population density) and some of the data and findings might not be transferable to other countries. Thirdly, due to the lack of GPS walking data, walking areas could only be delineated by buffer size. This could lead to an overestimation of walking times for older people. Fourthly, due to the lack of streetscape data for low-density neighbourhoods, we used the latest NDVI data and tried to reduce the amount of error by increasing the resolution of the NDVI to 10 m to ensure accuracy, but some unusable green space was still accounted for. This was expected to be addressed by the subsequent addition of street view data. Finally, all streets in Guangzhou were walkable, but there were a few expressways that were included, which we believe would have minimal impact on our model.

## Conclusions

In this study, we examined the differences between the residential environments of older people in Guangzhou at different densities in relation to recreational and transport walking, using a more comprehensive sample of data that provides credible evidence that low, medium, and high densities are different from each other. The environmental characteristics of the medium density areas correlated with both recreational walking and transport walking, while high and low densities did not. The correlation between various environmental characteristics (e.g., land use diversity) and walking in different buffer zones in medium density areas was also significant, indicating that medium density environment might play a more significant role in affecting physical activity, including walking among older residents than other densities. Our findings might be of better help to cities and older people for studies in cities of different densities in the future.

## Data Availability Statement

The raw data supporting the conclusions of this article will be made available by the authors, without undue reservation.

## Author Contributions

PZ: conceptualisation. SM and FX: resources. XZ and YZ: supervision. HQ: validation. PZ and HQ: writing—original draft. PZ: writing—review and editing. All authors contributed to the article and approved the submitted version.

## Funding

This research was funded by National Natural Science Foundation of China (No. 51908135) and Guangdong Office of Philosophy and Social Science (No. GD20CSH04).

## Conflict of Interest

The authors declare that the research was conducted in the absence of any commercial or financial relationships that could be construed as a potential conflict of interest.

## Publisher's Note

All claims expressed in this article are solely those of the authors and do not necessarily represent those of their affiliated organizations, or those of the publisher, the editors and the reviewers. Any product that may be evaluated in this article, or claim that may be made by its manufacturer, is not guaranteed or endorsed by the publisher.

## Footnotes

^1^Density Zone 1 (≥10,000 persons/km2): within the city ring road, the centre of Shiqiao Town in Panyu District, the centre of Xinhua Town in Huadu District; Density Zone 2 (5,000-10,000 persons/km2): south of Guangyuan Road, the Tianhe section of South China Avenue, west of the Haizhu section of Guangzhou Avenue, north of Xinqiao South Road in Fangcun District, and from Fangcun Avenue to the Ruyifang Bridge in Hedong. Huangpu District, south of Guangshen Railway, east of Maogang Road, west of Shihua Road, east of the bridge section north of Gangqian Road, and the sub-centre area of the city. Fan Shiqiao Town, Huadu District, Xinhua Town, Huadu District, Panyu District except Density 1, Luoxi, Dashi, Shilou, Li Town, Zengcheng, Conghua Street. The central area is the central area of the town of Kou; Density Zone 3 (≤ 5000 persons/km2). Zhiwei Tang area east of Huadi River in Fangcun District; Xinzhou, Chigang, and Nanzhou areas in Haizhu District; Tianhe District, Huangpu District, Huancheng Expressway-Guangzhou-Shenzhen Expressway South, Longdong area; Baiyun District, South China Avenue South, Jianggaocheng Street South; Central District. Dongchong and Lingshan Urban Sub-centres in Panyu District and Xinhua Urban Sub-centre in Huadu District.

^2^sDNA is the world's leading 2D and 3D spatial network analysis software for GIS, CAD, command line and Python, using industry standard network representations. We calculate accessibility and predict flows of pedestrians, cyclists, vehicles and public transport users; these inform models of health, community cohesion, land values, town centre vitality, land use, accidents and crime. We offer a simpler alternative to transport modelling, particularly for sustainable transport (34).
